# Differentially expressed genes in embryonic cardiac tissues of mice lacking *Folr1 *gene activity

**DOI:** 10.1186/1471-213X-7-128

**Published:** 2007-11-20

**Authors:** Huiping Zhu, Robert M Cabrera, Bogdan J Wlodarczyk, Daniel Bozinov, Deli Wang, Robert J Schwartz, Richard H Finnell

**Affiliations:** 1 Center for Environmental and Genetic Medicine, Institute of Biosciences and Technology, Texas A&M University System Health Science Center, Houston, Texas 77030, USA; 2 Center for Molecular Development and Diseases, Institute of Biosciences and Technology, Texas A&M University System Health Science Center, Houston, Texas 77030, USA; 3 Department of Pediatrics, UNMC, Omaha, NE 68158, USA; 4 Biostatistics and Bioinformatics Unit, Comprehensive Cancer Center, The University of Alabama at Birmingham, Birmingham, AL, 35294, USA

## Abstract

**Background:**

Heart anomalies are the most frequently observed among all human congenital defects. As with the situation for neural tube defects (NTDs), it has been demonstrated that women who use multivitamins containing folic acid peri-conceptionally have a reduced risk for delivering offspring with conotruncal heart defects [1-3]. Cellular folate transport is mediated by a receptor or binding protein and by an anionic transporter protein system. Defective function of the *Folr1 *(also known as *Folbp1*; homologue of human *FRα*) gene in mice results in inadequate transport, accumulation, or metabolism of folate during cardiovascular morphogenesis.

**Results:**

We have observed cardiovascular abnormalities including outflow tract and aortic arch arterial defects in genetically compromised *Folr1 *knockout mice. In order to investigate the molecular mechanisms underlying the failure to complete development of outflow tract and aortic arch arteries in the *Folr1 *knockout mouse model, we examined tissue-specific gene expression difference between *Folr1 *nullizygous embryos and morphologically normal heterozygous embryos during early cardiac development (14-somite stage), heart tube looping (28-somite stage), and outflow track septation (38-somite stage). Microarray analysis was performed as a primary screening, followed by investigation using quantitative real-time PCR assays. Gene ontology analysis highlighted the following ontology groups: cell migration, cell motility and localization of cells, structural constituent of cytoskeleton, cell-cell adhesion, oxidoreductase, protein folding and mRNA processing. This study provided preliminary data and suggested potential candidate genes for further description and investigation.

**Conclusion:**

The results suggested that *Folr1 *gene ablation and abnormal folate homeostasis altered gene expression in developing heart and conotruncal tissues. These changes affected normal cytoskeleton structures, cell migration and motility as well as cellular redox status, which may contribute to cardiovascular abnormalities in mouse embryos lacking *Folr1 *gene activity.

## Background

Heart defects account for nearly one-third of all major congenital anomalies diagnosed in fetuses and infants [4], but the etiologies of heart anomalies are largely unknown. Most heart anomalies are suspected of being etiologically and pathogeneticly heterogeneous [5]. Conotruncal defects are a group of defects which result from abnormal aortico-pulmonary septation of the outflow tract of the heart, a process that has been shown to have a major mesectodermal cell contribution [6-10]. Despite this understanding of the pathogenesis of conotruncal defects, little is actually known about the etiology of these heart defects.

Shaw and co-workers [1] observed a 30% risk reduction for conotruncal defects among the offspring of women who used multivitamins containing folic acid in early pregnancy. The risk reduction for the group was driven by a larger risk reduction for the Tetralogy of Fallot. Early studies found that folate deficiency during gestation were associated with multiple congenital abnormalities in rats, including those heart anomalies similar to conotruncal defects [11,12]. Additional evidence in support of the protective effect of folic acid comes from reports of an association between maternal anticonvulsant use and heart defects [13]. Most of the frontline anticonvulsants are known to be folate antagonists [14], and reduction in the bioavailability of folate to the fetus has been proposed as one of their potential underlying teratogenic mechanisms of action [15,16].

Several lines of evidence support an association between maternal use of folic acid in early pregnancy and a reduced risk for delivering offspring with conotruncal defects. However, the underlying process by which folic acid facilitates a reduction in risk is unknown and remains an area of considerable scientific speculation. Given the overall evidence that has emerged from the studies of a closely related set of congenital defects, the induction of conotruncal defects also is unlikely to be explained by a simple maternal vitamin deficiency. The evidence accumulated in recent years suggested that elevated homocysteine (Hcy) levels may be a major teratogenic mechanism underlying folic acid deficiency [17-19]. We hypothesize that a fetal deficiency in transport and/or metabolism of folate puts fetuses at risk for conotruncal defects, and that maternal folic acid supplementation helps overcome this deficiency. These defects are hypothesized to be the result of a direct effect of folate insufficiency on the growth and differentiation of embryonic cells. In addition, neural crest cells that contribute to conotruncal septation are rapidly dividing cells that require adequate intracellular folate supply that can best be facilitated by a well-regulated folate uptake pathway. We recently characterized the cardiovascular defects in *Folr1 *knockout mouse model [20]. Cardiac outflow tract defects, including double outflow right ventricle (DORV), rightward persistent truncus arteriosus (PTA) and transposition of great arteries (TGA) have been observed in pre-term nullizygotes rescued from lethality by low dose maternal folate supplementation. We also observed aortic arch arteries defects such as right aorta arch, aorta ring/double aorta and interrupted aorta arch in these fetuses [20].

We hypothesized that *Folr1 *gene ablation will alter the expression of other genes which may be important for normal cardiovascular development. These gene expression changes may affect biological functions of the developing heart, and ultimately result in one of several defects we observed in *Folr1 *mutant mice. In order to investigate the mechanisms of cardiovascular abnormalities induced by knocking out *Folr1 *gene, we designed experiments in which *Folr1 *heterozygous female were crossed to *Folr1 *nullizygous male mice. Pregnant dams were given low dose of s-folinic acid in order to rescue the nullizygote's embryonic lethality. We subsequently collected heart and conotruncal tissues, extracted total RNA and studied gene expression within isolated regions of the target tissues. We investigated the gene expression changes induced by conventional *Folr1 *gene ablation in the cardiac tissue in order to discover patterns that might shed light on the mechanisms of how *Folr1 *gene and folate status regulate early cardiac development.

## Results

### Embryos collected from s-folinic acid supplemented dams (Table 1)

**Table 1:** *Folr1 *nullizygous and heterozygous embryos collected from s-folinic acid supplemented dams

**Gestational Day (day:hour)**	**Dams (N)**	**Genotype**	**Embryos (n)**	**Somites (mean ± SD)**	**P value (Student's T-test)**
**9:00**	3	-/-	11	5.7 ± 2.7	<0.001
		+/-	17	12.5 ± 2.7	
**9:12**	5	-/-	20	12.8 ± 3.3	<0.001
		+/-	16	20.2 ± 5.9	
**10:00**	3	-/-	9	19.1 ± 3.1	<0.001
		+/-	12	28.1 ± 0.9	
**10:12**	5	-/-	16	28.3 ± 2.7	<0.001
		+/-	12	34.9 ± 3.3	
**11:12**	3	-/-	13	33.6 ± 6.4	= 0.07
		+/-	12	38.2 ± 5.4	

The average somite number of nullizygous embryos collected at E9.5 was 12.8 (± 3.3). Their heterozygous littermates developed faster and had an average somite number of 20.2 (± 5.9). Average somite numbers for embryos collected at E10.5 were 28.3 (± 2.7) for the nullizygous and 34.9 (± 3.3) for the heterozygous embryos. The somite number differences between mutants and heterozygotes at these two time points were statistically significant (Student's T-test, p < 0.001); therefore we were unable to obtain somite matched littermate controls. In order to match somites, we decided to collect heterozygous embryos at earlier time points (E9.0 and E10.0). Heterozygous embryos averaged 12.5 (± 2.7) somites at E9.0, and 28.1 (± 0.9) somites at E10.0, which matched the nullizygous embryos collected at E9.5 and E10.5, respectively. Knockout embryos collected at E11.5 also showed developmental delay; however, the somite number did not differ significantly from their heterozygous littermates (Student's T-test, p > 0.05) and we managed to collect comparable null and heterozygous embryos (Table 1).

### Microarray data

Microarray analyses were performed as preliminary screening for candidate genes. The original data from this study have been deposited in NCBI's Gene Expression Omnibus (GEO) under GEO Series Accession No. GSE3487. At the 14 somite stage, out of approximately 20,000 genes, 23 genes were down-regulated and 18 were up-regulated in cardiac tissue. Among these genes, six of the down-regulated genes and five of the up-regulated genes are unknown. At the 28 somite stage, 37 genes were down-regulated and 26 were up-regulated in cardiac tissue, with 15 being functionally unknown. At the 38 somite stage, out of approximately 10,500 genes, 27 genes were down-regulated and 16 genes were up-regulated in the conotruncal tissue, with 12 of them being functionally unknown. Analysis indicated that the *Cck *gene, encoding cholecystokinin, was down-regulated 1.6 fold in cardiac tissue from a 14 somite fetus, and decreased still further (2.8 fold) in tissue from older (28 somite) fetuses. This is the only gene showing differential expression in multiple time points. We further conducted gene ontology (GO) analysis with the use of GOTM [21]. The GO analysis produced clusters of statistically (p < 0.01) enriched differentially expressed genes according to their ontology in three categories: biological processes, cellular component and molecular function. The ontology groups enriched at each developmental stage are shown in Table 2.

**Table 2:** Gene Enrichment analysis of developing heart and outflow track in *FolR1*^-/- ^embryos (P < 0.01)

**Gene Ontology category**	**Ratio of Enrichment (R)**
**14-somites heart**	
Cell migration	9.09
Cell motility	7.69
Localization of cells	7.69
mRNA processing	9.38
RNA binding	5.26
**28-somites heart**	
Structural Constituent of cytoskeleton	9.68
Translation regulation activity	7.32
Translation factor activity/nucleic acid binding	7.69
mRNA processing	5.8
RNA polymerase transcription factor activity	14.29
**38-somites conotruncal tissue**	
Oxidoreductase activity	3.73
Protein folding	7.14
Intracellular membrane-bound organelle	9.64
Nuclear membrane	21.43
**All changed genes**	
Cell-cell adhesion	5.13
mRNA processing	5.74
Intracellular membrane-bound organelle	32.61

### Quantitative real-time PCR (qRT-PCR)

We performed quantitative RT-PCR using TaqMan Gene Expression Assays (Applied Biosystems, Foster City, CA) on eight candidate genes suggested by the preliminary microarray data. The candidate genes we studied included *Mylpf*, *Cck*, *Cfl1 *and *Nkd2 *(14-somite stage), *Cck *and *Hand1 *(28-somite stage), *Fbln5*, *Capns1 *and *Canx genes *(38-somite stage). Tables 3 summarized the comparison of gene expression data obtained from quantitative RT-PCR and microarray. For each gene tested, the qRT-PCR result is consistent with the microarray result; *Mylpf, Cck, Cfl1 *and *Fbln5 *were down-regulated, while *Nkd2, Hand1, Capns1 *and *Canx *were up-regulated in nullizygous tissue samples compared to control samples. The fold changes obtained from microarray data was also comparable to those from qRT-PCR assays. However, qRT-PCR changes in *Nkd2 *and *Fbln5 *were not statistically significant (P > 0.05). We subsequently expanded the analysis of *Cfl1*, *Cck *and *Hand1 *genes to all three stages. qRT-PCR results showed that *Cfl1 *was only down-regulated in 14-somite stage (p = 0.002) but not in 28-somite heart or 38-somite conotruncal tissue (p > 0.05). *Cck *was about 2 fold down-regulated (p = 0.004) in 14-somite heart, 4.5 fold down-regulated in 28-somite heart (p = 0.005), and 1.7 fold down-regulated in 38-somite conotruncal tissue, however the change is not statistically significant (p = 0.30) (Table 3). *Hand1 *is unchanged at 14-somite stage, 2 fold up-regulated in 28-somite stage (p = 0.002) heart, but down-regulated in 38-somite conotruncal tissue, however, the change is not statistically significant (p = 0.07).

**Table 3:** Gene expression changes in *Folr1*^-/- ^heart and conotruncal tissue-qRT-PCR

**Gene symbol**	**Gene Name**	**Ontology**	**qRT-PCR**					
			14-somite		28-somite		38-somite	
			Fold change	*p value	Fold change	*p value	Fold change	*p value

*Mylpf*	myosin light chain, phosphorylatable, fast skeletal muscle	cytoskeleton organization and biogenesis	**-2.8**	**0.006**	---	---	---	---
*Cfl1*	cofilin 1, non-muscle	Neural crest cell migration	**-1.7**	**0.002**	-1.1	0.3	-1.1	0.24
*Nkd2*	naked cuticle 2 homolog (Drosophila)	Wnt signaling pathway	**1.6**	**0.449**	---	---	---	---
*Cck*	cholecystokinin	Cell migration	**-1.9**	**0.004**	**-4.5**	**0.005**	-1.7	0.30
*Hand1*	Heart and neural crest derivatives expressed transcript 1	transcription regulation, heart development, angiogenesis	1.0	0.444	**+2.0**	**0.002**	-1.8	0.07
*Fbln5*	Fibulin-5	Cell-cell adhesion	---	---	---	---	**-7.1**	0.098
*Capns1*	Calpain, small subunit 1 (Capns1)	calpain activity	---	---	---	---	**+7.4**	**0.001**
*Canx*	Calnexin	Protein folding	---	---	---	---	**+7.6**	**0.036**

*One-side T-test

## Discussion

Our study was designed to investigate differentially expressed genes related to the heart and conotruncal phenotypes in response to conventional knockout of mouse *Folr1 *gene. We used "het ♀ × null ♂" matings to generate heterozygous and nullizygous progeny. It has been established that *Folr1 *heterozygotes do not exhibit any morphological phenotypes, although their biochemical status may differ from wild type due to the loss of one *Folr1 *allele [22]. The nullizygous males used in the mating were born from colonies maintained on a high folate diet, therefore these mutant animals were completely rescued from *Folr1 *ablation related morphological phenotypes. The heterozygous embryos served as controls in the micorarray studies under the same maternal supplementation regime as the nullizygotes. Embryos used for study were matched by somite number which represents gross developmental progress. We chose not to use littermate matches, since the nullizygotes under the folate supplementation regime we used were significantly delayed compared to heterozygous littermates (Table 1).

Early embryonic heart development (E9.0 to E11.5) undergoes a series of highly complex, coordinated and rapid morphogenesis processes. In *Folr1 *mutant embryos provided in utero with low dose of maternal folate supplementation (6.25 mg/kg/day s-folinic acid), abnormal heart looping including inverted looping, midline looping, and shorterned outflow tract was observed as early as E10.0 in previous experiments. Abnormal looping in these animals contributes to the mis-alignment of outflow tract and some phenotypes seen in the pre-term human fetuses. Our experiments took snap shots of the gene expression patterns at two different stages (14-somite and 28-somite) in heart tissues, and later in more restricted conotruncal tissues (38-somite). The data suggests that changes in gene expression are responsible, at least in part, for the observed cardiac malformations.

**Figure 1:**
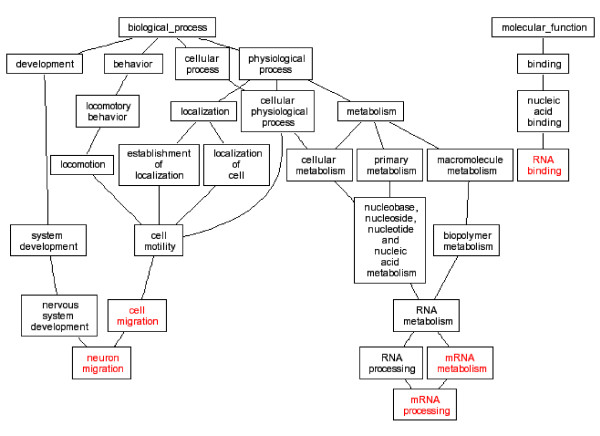
**DAG view of Gene ontology Analysis, 14-somite heart tissue in *Folr1 *mutant vs control. Red letters indicated enriched ontology groups**. **DAG: **Directed Acyclic Graph.

**Figure 2:**
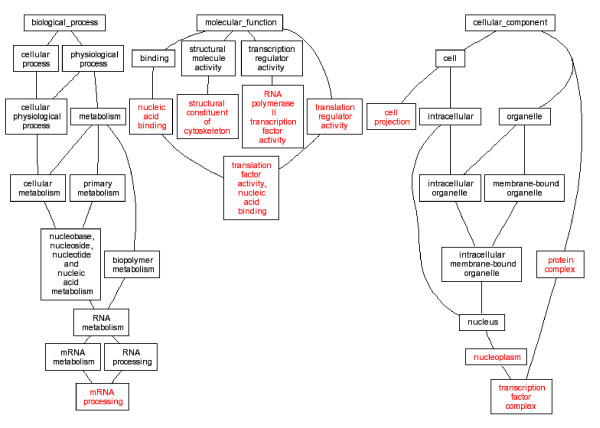
DAG view of Gene ontology Analysis, 28-somite heart tissue in *Folr1 *mutant vs control. Red letters indicated enriched ontology groups.

**Figure 3:**
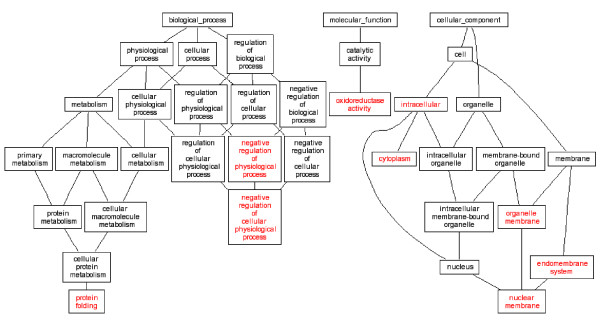
DAG view of Gene ontology Analysis, 38-somite conotruncal tissue in *Folr1 *mutant vs control. Red letters indicated enriched ontology groups.

**Figure 4:**
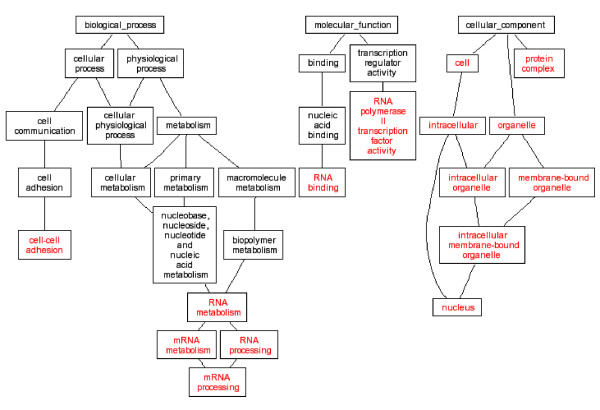
DAG view of Gene ontology Analysis, All changed genes in *Folr1 *mutant vs control. Red letters indicated enriched ontology groups.

It is generally hypothesized that multiple genes and pathways are responsible for complex birth defects. Gene expression alterations in the embryonic tissues ultimately contributed to these malformations. Our study produced lists of up and down regulated genes, and these genes were classified by their gene ontologies. GO analysis of our microarray data highlighted several ontology groups which were most significantly enriched in *Folr1 *mutant heart and conotruncal tissues (Table 2, Figures 1, 2, 3, 4). We further investigated eight candidate genes selected based on the microarray data.

Embryonic heart development is an extremely complex process requiring highly organized and coordinated cell movement. The actin cytoskeleton is intimately involved in regulating cell motility, membrane trafficking, cell polarity and signal transduction. During early heart development in *Folr1 *mutant embryos, the expression of a number of structural cytoskeleton genes were altered. These included *Actb*, encoding the "house-keeping" cytoplasmic beta-actin (down-regulated in 28-somite heart tissue), *Arpc5*, encoding Actin related protein 2/3 complex subunit 5 (down-regulated in 28-somite heart tissue) and *Actl7a*, encoding Actin-like 7a (up-regulated in 28-somite heart tissue). ***Mylpf ***(phosphorylatable myosin light chain, fast skeleton muscle, previously named as "myosin light chain 2a, *Mlc2a*"), was found to be down-regulated in 14-somite tubular heart tissue. *Mylpf *expression in tubular heart exhibits a gradient, while in later stages, *Mylpf *is expressed at high levels in the outflow tract, atria, and inflow tract [23]. Cholecystokinin, encoded by ***Cck***, is known as a brain/gut peptide, whose functional roles remain unclear. There is evidence showing that *Cck *may involve in development of neurons [24] by modulating cell migration. We observed down regulation of *Cck *in both 14-somite and 28-somite hearts. Further investigation of *Cck *and cardiac development is warranted.

Actin-based movement results from rapid turnover of active filaments which requires Arp2/3 complex, actin depolymerizing factor and capping proteins [25]. ***Cfl1***, encoding non-muscle cofilin (n-cofilin), was down-regulated in 14-somite heart tissue. n-cofilin is an actin-depolymerizing factor and is essential for cytokinesis, endocytosis, and in the development of all embryonic tissues. *Cfl1 *knockout mice exhibit failure of neural tube closure at E10.5 and die *in utero*. In these embryos, the delamination and migration of neural crest cell is inefficient. *In vitro *migration assay showed no signs of cell polarization, limited traveling distance and lack of F-actin structures (fibers, bundles or cortical F-actins) [26]. Reduced *Cfl1 *expression, together with later reduced expression of *Actb *and *Arpc5 *in *Folr1 *mutant heart tissues could contribute to abnormal actin dynamic, cell polarity and cell migration in these embryos.

The expression changes in the aforementioned cytoskeletal related genes in cardiac tissues of *Folr1 *knockout mice during different stages of cardiac development support the hypothesis of possible impairment of cytoskeletal structure and cell motility of neural crest, myocardial and endocardial cells. Another study found that genes involved in the semaphorin/plexin signaling pathway which regulates cofilin and actin cytoskeleton [27] were differentially expressed in E12.5 heart tissue from *Folr1 *knockout mice (Gelineau-van Waes et al., Submitted). Such altered gene expression may subsequently contribute to the ultimate cardiac phenotypes observed in the mutant mice.

Calpain is a Ca^2+^-regulated cytosolic cysteine protease that exists in two major isoforms and mediates crucial cellular functions including rearrangement of cytoskeletal proteins and protein cleavage to activate various receptors and pro-enzymes. Calpain protein consist of a large activity subunit and a small regulatory subunit. Calpain dysregulation results in a loss of Ca^2+ ^homeostasis and intracellular calpain activation, leading to degradation of a large family of calpain-specific substrates and physiologically induces tissue damage. Cellular proteins including cytoskeletal proteins, membrane receptors (e.g. epidermal growth factor (EGF) and G proteins), signaling molecules (e.g. integrin, protein kinase C and inositol (1,4,5)-trisphosphate kinase), and transcriptional factors (e.g. c-FOS and c-JUN) have all been identified as potential calpain substrates [28]. Calpain related pathology seems to be of enormous diversity [29]. Calpain is known to modulate actin cytoskeleton and cell migration by regulating activities of signaling molecules including integrin, focal adhesion kinase, talin, protein kinase C and the *Rho *family of GTPase [30]. The ***Capns1 ***gene encodes the small subunit of μ- and m-calpains, which is known to be essential for embryonic development [31]. Genetic ablation of the calpain small subunit exhibited abnormal neural crest cell migration [32]. We observed a significant increase in *Capns1 *expression in *Folr1 *mutant conotruncal tissues at the 38-somite stage. At this stage, the outflow tract is being remodeled to form endocardium cushions and develop into the aorta and pulmonary artery. The observed up-regulation of *Capns1 *suggests a possible dysregulation of calpain activity and the loss of Ca^2+ ^homeostasis. It remains unclear which genes are serving as down-stream targets of calpain in our mouse model. It is also possible that an increase of the small subunit is actually a feedback response to intracellular oxidative stress caused by the *Folr1 *gene ablation and/or folate deficiency.

Cell-cell interaction is a fundamental process required for mammalian development. Cells interact with each other through cell adhesion. Cadherins are a group of cell adhesion proteins that mediate Ca^2+^-dependent cell-cell adhesion. The functional significance of these proteins during embryogenesis has been previously revealed [33]. The members of the cadherin superfamily (cadherins) are characterized by their unique extracellular domains composed of multiple cadherin repeats. Classical cadherins, such as epithelial (E-) or neuronal (N-) cadherin, link to the cytoskeleton to establish strong adhesion. This is mediated by binding of the conserved cytoplamic tail to β-catenin. The cadherin-β-catenin complex then binds to α-catenin which bridges the complex to the actin cytoskeleton via actin-binding proteins such as α-actinin or profilin [34]. Interestingly, β-catenin is also a key player in the canonical Wnt signaling pathway, suggesting important interrelations between Wnt signaling and cadherin-mediated adhesion [35].

Wnts are a group of important extracellular glycoproteins. Wnt signaling plays critical roles in many biological processes such as regulation of cell adhesion, cell proliferation, differentiation and transcription of target genes. Recent studies from different species suggested Wnt signaling is also involved in cardiac development [36]. Wnt11 is a key regulator of cardiac muscle cell proliferation and differentiation during heart development [37]. Canonical Wnt signaling is required for proper cardiac differentiation [38] and neural crest cell induction, while non-canonical Wnt pathways (Wnt/PCP and Wnt-Ca^2+^) are essential for neural crest migration [39]. ***Nkd2***, naked cuticle 2 homolog (Drosophila), encodes NKD2, which is a calcium binding protein known to bind an important signaling molecule, *Dishevelled*, and antagonizes both canonical Wnt signaling and PCP pathway [40,41]. During mouse embryo development, *Nkd1 *and *Nkd2 *are expressed in multiple tissues in partially overlapping, gradient-like pattern, some of which correlate with known patterns of Wnt activity. Increased *Nkd2 *expression in 14-somite *Folr1 *mutant heart tissues may inhibit *Wnt-Dishevelled *signaling pathways in these embryos and contribute to abnormal cardiac development at this stage.

***Canx ***(Calnexin) was up-regulated in 38-somite conotruncal tissues). The calnexin protein is an important component of the calreticulin/calnexin cycle and the quality control pathways in the ER. Disruption of this cycle may cause impaired cardiac development [42,43]. These may reflect complex changes of cell-cell and cell-matrix interaction which affect cell behaviors such as polarity and motility. Other genes related to cell adhesion and ECM found to be differentially expressed in *Folr1 *mutant heart and conotruncal tissues included ***Fbln5 ***(Fibulin-5, up-regulated in 38-somite conotruncal tissue), *Aplp2 *(Amyloid beta precursor-like protein 2, down-regulated in 28-somite heart tissues), *Bbp *(Beta-amyloid binding protein precursor, down-regulated in 28-somite heart tissues), *Cldn18 *(Claudin 18, up-regulated in 38-somite conotruncal tissues), *Nr2f2 *(Nuclear receptor subfamily 2, group F, member 2, down-regulated in 14-somite heart tissues) and *Col4a3bp *(Procollagen, type IV, alpha 3 binding protein, down-regulated in 28-somite heart tissues).

The bHLH transcription factor, ***Hand1***, plays an important role in cardiac morphogenesis. *Hand1 *has been identified a crucial cardiac regulatory protein that controls the balance between proliferation and differentiation in the developing heart [44]. HAND1 protein acts as cell-specific developmental co-activators of the MEF2 family of transcription factors [45]. We observed an increase of *Hand1 *expression in 28-somite heart, suggesting possible involvement of *Hand1 *in cardiac phenotype in *Folr*^-/- ^mice. In 38-somite conotruncal tissue, however, *Hand1 *was down-regulated. Further investigation is needed to characterize the expression of *Hand1 *in *Folr*^-/- ^embryos.

Our microarra data also showed that a group of genes involved in oxidoreductive reactions were changed in *Folr1 *mutants. These included: Peroxiredoxin (*Prdx1*, down-regulated in 28-somite heart;*Prdx2*, down-regulated in 38-somite conotruncal tissue), Glutathione S-transferase, mu5 (*Gstm5*, down-regulated in 28-somite heart), Coproporphyrinogen oxidase (*Cpox*, down-regulated in 14-somite heart), Phosphogluconate dehydrogenase (*Pgd*, down-regulated in 14-somite heart), ATPase, H+ transporting, V0 subunit (*Atp6v0e*, down-regulated in 38-somate conotruncal tissue), Lactate dehydrogenase 2, B chain (*Ldh2*, down-regulated in 38-somate conotruncal tissue), Acetyl-Coenzyme A dehydrogenase, long-chain (*Acadl*, down-regulated in 38-somate conotruncal tissue), Ribosomal protein L4 (*Rpl4*, down-regulated in 38-somate conotruncal tissue), Cytochrome P450, family 2, subfamily b, polypeptide 19 (*Cyp2b19*, up-regulated in 38-somate conotruncal tissue), and Serine/threonine kinase 11 interacting protein (*Stk11ip*, up-regulated in 38-somate conotruncal tissue).

Oxidative stress is involved in the etiology of a spectrum of diseases including those of the cardiovascular diseases, birth defects, immune diseases, and cancer. Increased generation of ROS or impaired ROS scavenging function also play a central role in a variety of teratogenic processes, such as maternal diabetes/obesity, environmental (arsenic) and drug-induced teratogenesis [46,47]. Folr1 knockout mice are likely to suffer from oxidative stress secondary to disturbed folate homeostasis. Changes of genes involved in the generation of ROS and/or RNS and antioxidant defense machinery observed in this study provided supportive evidence to the hypothesis that oxidative stress contributes significantly to abnormal cardiovascular development and myocardial function under a rather complicated mechanism.

Even though the gene ontology analysis of microarray data is limited to current literature and knowledge, it provided important clues for generation of new testable hypotheses. Further studies focused on pathway-specific gene expression, proteomic and functional validation of candidate genes, as well as interactions among responsive genes and pathways, are currently underway in our laboratory.

The exploratory nature and small sample size (three in each group) of the microarray study resulted in limited power of statistical tests, which may subsequently cause excessive false negative results. Control of false discovery rate (FDR) has become popular in microarray data analysis [48]. For our study specifically, however, FDR correction does not help to identify differentially expressed genes (data not shown). We therefore used combined criteria to select candidate genes for further study: 1) at least 1.5 fold changes in gene expression; 2) t-test p-value < 0.05; 3) average intensity above background signal plus four standard deviations. This criterion is suitable for using microarray data as preliminary screening. Selected genes were further investigated using standard quantitative real-time PCR technology.

## Conclusion

The results suggested that *Folr1 *gene ablation and abnormal folate homeostasis altered gene expression in developing heart and conotruncal tissues. These changes affected normal cytoskeleton structures, cell migration and motility as well as cellular redox status, which contributed to cardiovascular abnormalities in mouse embryos lacking *Folr1 *gene activity.

## Methods

### Animal husbandry

All mice were housed in clear polycarbonate micro-isolator cages, allowed free access to water and food, and were maintained on a 12-hr light/dark cycle in the Vivarium at the Institute of Biosciences and Technology in Houston, Texas. *Folr1*-deficient mice were generated by standard gene targeting methodologies [49]. *Folr1 *heterozygous mice were transferred to the highly inbred LM/Bc genetic background (*L-Folr1*), and were maintained by brother-sister matings for at least 10 generations. Considering the embryonic lethality of the *Folr1 *knockout, breeders were maintained on a modified Clifford/Koury folate deficient diet supplemented with 200 mg/kg folic acid and succinyl sulfathiazole (Dyets Inc., Bethlehem, PA), in order to obtain viable nullizygous individuals. Heterozygous and nullizygous embryos were generated by timed-matings between *L-Folr1 *heterozygous females and nullizygous males maintained on a normal diet. The day on which vaginal plug was found was designated as E0.5.

### Experimental design

Embryos from three different gestational ages: 14-somite (E9.0~9.5), 28-somite (E10.0~10.5) and 38-somite (11.5) were harvested in order to perform gene expression comparisons. At 14-somite stage, the heart tube was dissected out from the arterial end to the venous end. At the 28-somite stage, the outflow tract, ventricle chambers and atrial chambers were dissected out. At the 38-somite stage, only the outflow tract (conotruncal tissue) was collected. Heterozygous control samples were chosen by matching somite numbers with the nullizygous samples. Six embryonic tissue samples from separate dams (three mutants and three controls) for each time point were used for microarray analyses. Ten embryonic tissue samples from separate dams (five mutants and five controls) were collected for Q-PCR analyses, and triplicate assays were used for each RNA specimen. RNA from all samples were isolated and assayed individually without pooling.

### Genotyping

Genomic DNA was extracted from yolk sac tissue using Puregene DNA extraction Kit (Gentra, Minneapolis, MN). Exon2 was amplified using primer pair: 5'-AATGTCAAGGCTGCATGTGG-3' and 5'-CATTCCGATGTCATAGTTCCGC-3' to detect wild type *Folr1*; the neo cassette was amplified using primer pair: 5'-CTTGGGTGGAGAGGCTATTC-3' and 5'-TGCATTCCGATGTCATAGTTCCG-3' for the identification of the mutant *Folr1 *allele. The PCR condition included an initial denaturation at 95°C for 5 min, followed by 30 cycles of denaturation (95°C for 1 min), annealing (60°C for 1 min) and extension (72°C for 2 min) and a final extension at 72°C for 10 min. PCR product was examined on 2% agarose gel under UV light [49]. The 179 BP or 1.2 kb products corresponded to the wild type and mutant alleles, respectively.

### Tissue collection and RNA preparation

Pregnant dams maintained on the normal diet were treated p.o. with 6.25 mg/kg (6 s) 5-Formyl H_4 _folate (s-folinic acid) from E0.5, in order to rescue the embryonic lethality. This treatment condition was chosen because in our previous experiments, the same level of supplementation rescued 80% of the nullizygous embryos from embryonic death when examined on E 11.5, although more than 90% of surviving nullizygous embryos presented with cardiovascular abnormalities. The pregnant dams were sacrificed by cervical dislocation and the fetuses were dissected immediately free of maternal deciduas in cold RNase-free PBS solution to minimize loss and change of mRNA. 14-somite stage heart tube, 28-somite stage heart and 38-somite stage conotruncal tissue of *Folr1 *nullizygous and heterozygous embryos were collected and stored in RNA *later*-ICE (Ambion, Austin, TX) in -80°C until needed for the RNA preparation. Total RNA was extracted using PicoPure RNA Isolation Kit (Acturus, Mountain View, CA) following the manufacturer's protocol.

### cDNA synthesis and aRNA amplification

In order to obtain sufficient RNA for our microarray experiments, we performed antisense RNA (aRNA) amplification using total RNA extracted from embryonic tissues. Oligo dT_24_-V-T7 primer (Ambion, Austin, TX) were added during the synthesis of double strand cDNA. Two rounds of aRNA amplification were performed using MEGAscript kit (Ambion, Austin, TX). aRNA were subsequently purified using Qiagen RNeasy kit and the aRNA quantity was determined using a fluorometer and the fluorescent nucleic acid stain RiboGreen (Molecular Probes).

### Microarray assay

Gene expression analysis was performed using CodeLink mouse genome Bioarrays. The CodeLink UniSet Mouse 20 K ((GE Healthcare, Piscataway, NJ) arrays were used for 14-somite and 28-somite heart tissue samples. For conotruncal tissue samples, the CodeLink UniSet Mouse I (10 K, ~10,500 genes) arrays were used. These arrays have the ability to detect a 1.3 fold change in gene expression with 95% confidence or 2-fold with 98% confidence while differentiating between targets (Codeline Bioarrays; GE Healthcare, Piscataway, NJ). 10 μg of aRNA was fragmented in 40 mM Tris acetate, pH 7.9, 100 mM KOAc and 31.5 mM MgOAc, at 94°C for 20 minutes. Hybridization, coupling of Alexa Fluor 647-streptavidin, and subsequent washes were performed according to manufacturer's protocol (GE Healthcare, Piscataway, NJ). The aRNA was mixed with buffer component A and B and then denatured at 90°C for 5 minutes. Hybridization was allowed to go 18 hours at 37°C, while shaking at 300 rpm. Each slide was rinsed in TNT buffer (0.1 M Tris-HCl pH 7.6, 0.15 M NaCl, 0.05% Tween-20) at room temperature, followed by a wash at 42°C for 1 hour. Coupling of a biotin labeled hybridized probe to dye labeled-streptavidin was performed in a 1:500 dilution of Alexa Fluor 647-streptavidin. Slides were rinsed in deionized water, spun-dry, and then scanned using an Agilent DNA Microarray Scanner (Palo Alto, CA). Initial feature extraction from the images was performed using CodeLink Expression Analysis software v4.0 (GE Healthcare, Piscataway, NJ). Intensities for each individual gene were determined by the median intensity of all pixels within the spot's region. Subtraction of the median local background (computed from the subset of remaining pixels of the bounding box) yielded net intensities representing relative gene expression levels. Criteria for differential expression was set as 1) at least 1.5 fold increase/decrease in gene expression; 2) t-test p < 0.05 when comparing controls vs. nulls to ensure a consistent expression throughout the replicated arrays; 3) Average intensity above background signal plus four standard deviations. This threshold was set to eliminate very weakly expressed genes. Data Clustering analysis was performed using Hierarchical Clustering Explorer version 3.0 (Human Computer Interaction Laboratory, University of Maryland, College Park) software, and the parameters were set for average linkage using the Unweighted Pair Group Method with Arithmetic Mean (UPGMA) and Pearson Correlation Coefficient.

### Quantitative reverse-transcription PCR

TaqMan^® ^Gene Expression Assays were used to determine gene expression changes for selected candidate genes. Gene-specific probes and primer sets were purchased from Applied Biosystems (Foster City, CA). The assays were performed according to manufacturer's protocol on an ABI PRISM^® ^7900 HT Sequence Detection System (Applied Biosystems, Foster City, CA). Data was analyzed using SDS software v2.1 (Applied Biosystems, Foster City, CA). Mouse *Gapdh *gene was used as house-keeping control for quantitative RT-PCR because it exhibited consistent normalized intensity across all arrays. Relative standard curve method was used to generate quantitative values. Each reaction was replicated three times and the normalized mean value was used in the final comparisons. The level of gene expression was compared between *Folr1 *nullizygous tissues and control tissues, while an unpaired T-test was applied with critical P value set at 0.05.

### Gene ontology analysis

Gene ontology analysis was performed using Gene Ontology Tree Machine (GOTM), University of Tennessee and Oak Ridge National Laboratory) [21,50]. GOTM compares the distribution of interesting gene set in each GO category to those in the reference gene set and reports those enrichments that are statistically significant as determined by the hypergeometric test (P < 0.01). Ratio of enrichment is calculated as:

R=k/Kn/N
 MathType@MTEF@5@5@+=feaafiart1ev1aaatCvAUfKttLearuWrP9MDH5MBPbIqV92AaeXatLxBI9gBaebbnrfifHhDYfgasaacPC6xNi=xI8qiVKYPFjYdHaVhbbf9v8qqaqFr0xc9vqFj0dXdbba91qpepeI8k8fiI+fsY=rqGqVepae9pg0db9vqaiVgFr0xfr=xfr=xc9adbaqaaeGacaGaaiaabeqaaeqabiWaaaGcbaGaemOuaiLaeyypa0tcfa4aaSaaaeaacqWGRbWAcqGGVaWlcqWGlbWsaeaacqWGUbGBcqGGVaWlcqWGobGtaaaaaa@35C6@

***N***: *number of genes on array **n**: number of interesting genes*

***K***: *number of genes in given category on array **k**: number of interesting genes in given category*

The significance of gene enrichment in a given GO category is determined by:

p=∑i=kn(N−Kn−i)(Ki)(Nn)
 MathType@MTEF@5@5@+=feaafiart1ev1aaatCvAUfKttLearuWrP9MDH5MBPbIqV92AaeXatLxBI9gBaebbnrfifHhDYfgasaacPC6xNi=xI8qiVKYPFjYdHaVhbbf9v8qqaqFr0xc9vqFj0dXdbba91qpepeI8k8fiI+fsY=rqGqVepae9pg0db9vqaiVgFr0xfr=xfr=xc9adbaqaaeGacaGaaiaabeqaaeqabiWaaaGcbaGaemiCaaNaeyypa0ZaaabCaKqbagaadaWcaaqaamaabmaabaqbaeqabiqaaaqaaiabd6eaojabgkHiTiabdUealbqaaiabd6gaUjabgkHiTiabdMgaPbaaaiaawIcacaGLPaaadaqadaqaauaabeqaceaaaeaacqWGlbWsaeaacqWGPbqAaaaacaGLOaGaayzkaaaabaWaaeWaaeaafaqabeGabaaabaGaemOta4eabaGaemOBa4gaaaGaayjkaiaawMcaaaaaaSqaaiabdMgaPjabg2da9iabdUgaRbqaaiabd6gaUbqdcqGHris5aaaa@4739@

## Authors' contributions

HZ performed experiment design, animal management, sample collection, genotyping, RNA/cDNA preparation, realtime PCR, data analysis, gene ontology analysis and manuscript writing; RC performed cDNA preparation, microarray assay, microarray data extraction and gene ontology analysis; BW performed experiment design, animal managenment, sample collection, data analysis and manuscript writing; DB performed microarray data analysis; DW provided statistical analysis and statistical consultation; RS provided advices on experiment design and manuscript writing; RHF is the princaple investigator of the funding grant and provided advices on experiment design and manuscript writing. All authors read and approved the final manuscript.

## References

[B1] Shaw GM, O'Malley CD, Wasserman CR, Tolarova MM, Lammer EJ (1995). Maternal periconceptional use of multivitamins and reduced risk for conotruncal heart defects and limb deficiencies among offspring. Am J Med Genet.

[B2] Botto LD, Mulinare J, Erickson JD (2003). Do multivitamin or folic acid supplements reduce the risk for congenital heart defects? Evidence and gaps. Am J Med Genet A.

[B3] Botto LD, Olney RS, Erickson JD (2004). Vitamin supplements and the risk for  congenital anomalies other than neural tube defects. Am J Med Genet C Semin Med  Genet.

[B4] Zierler S, Rothman KJ (1985). Congenital heart disease in relation to maternal use of Bendectin and other drugs in early pregnancy. N Engl J Med.

[B5] Ferencz C, Boughman JA (1993). Congenital heart disease in adolescents and adults. Teratology, genetics, and recurrence risks. Cardiol Clin.

[B6] Kirby ML, Gale TF, Stewart DE (1983). Neural crest cells contribute to normal aorticopulmonary septation. Science.

[B7] Kirby ML, Stewart DE (1983). Neural crest origin of cardiac ganglion cells in the chick embryo: identification and extirpation. Dev Biol.

[B8] Kirby ML, Bockman DE (1984). Neural crest and normal development: a new perspective. Anat Rec.

[B9] Kirby ML (1990). Alteration of cardiogenesis after neural crest ablation. Ann N Y Acad Sci.

[B10] Kirby ML, Waldo KL (1990). Role of neural crest in congenital heart disease. Circulation.

[B11] Baird CD, Nelson MM, Monie IW, Evans HM (1954). Congenital cardiovascular anomalies induced by pteroylglutamic acid deficiency during gestation in the rat. Circ Res.

[B12] Nelson MM, Baird CD, Wright HV, Evans HM (1956). Multiple congenital abnormalities in the rat resulting from riboflavin deficiency induced by the antimetabolite galactoflavin. J Nutr.

[B13] Anderson RC (1976). Cardiac defects in children of mothers receiving anticonvulsant therapy during pregnancy. J Pediatr.

[B14] Dansky LV, Finnell RH (1991). Parental epilepsy, anticonvulsant drugs, and reproductive outcome: epidemiologic and experimental findings spanning three decades; 2: Human studies. Reprod Toxicol.

[B15] Wegner C, Nau H (1991). Diurnal variation of folate concentrations in mouse embryo and plasma: the protective effect of folinic acid on valproic-acid-induced teratogenicity is time dependent. Reprod Toxicol.

[B16] Wegner C, Nau H (1992). Alteration of embryonic folate metabolism by valproic acid during organogenesis: implications for mechanism of teratogenesis. Neurology.

[B17] Steegers-Theunissen RP, Boers GH, Trijbels FJ, Finkelstein JD, Blom HJ, Thomas CM, Borm GF, Wouters MG, Eskes TK (1994). Maternal hyperhomocysteinemia: a risk factor for neural-tube defects?. Metabolism.

[B18] Rosenquist TH, Ratashak SA, Selhub J (1996). Homocysteine induces congenital defects of the heart and neural tube: effect of folic acid. Proc Natl Acad Sci USA.

[B19] Kapusta L, Haagmans ML, Steegers EA, Cuypers MH, Blom HJ, Eskes TK (1999). Congenital heart defects and maternal derangement of homocysteine metabolism. J Pediatr.

[B20] Zhu H, Wlodarczyk BJ, Scott M, Yu W, Merriweather M, Gelineau-van Waes J, Schwartz RJ, Finnell RH (2007). Cardiovascular abnormalities in Folr1 knockout mice and folate rescue. Birth Defects Res A Clin Mol Teratol.

[B21] Zhang B, Schmoyer D, Kirov S, Snoddy J (2004). GOTree Machine (GOTM): a web-based platform for interpreting sets of interesting genes using Gene Ontology hierarchies. BMC Bioinformatics.

[B22] Spiegelstein O, Mitchell LE, Merriweather MY, Wicker NJ, Zhang Q, Lammer EJ, Finnell RH (2004). Embryonic development of folate binding protein-1 (Folbp1) knockout mice: Effects of the chemical form, dose, and timing of maternal folate supplementation. Dev Dyn.

[B23] Franco D, Markman MM, Wagenaar GT, Ya J, Lamers WH, Moorman AF (1999). Myosin light chain 2a and 2v identifies the embryonic outflow tract myocardium in the developing rodent heart. Anat Rec.

[B24] Giacobini P, Kopin AS, Beart PM, Mercer LD, Fasolo A, Wray S (2004). Cholecystokinin modulates migration of gonadotropin-releasing hormone-1 neurons. J Neurosci.

[B25] Loisel TP, Boujemaa R, Pantaloni D, Carlier MF (1999). Reconstitution of actin-based motility of Listeria and Shigella using pure proteins. Nature.

[B26] Gurniak CB, Perlas E, Witke W (2005). The actin depolymerizing factor n-cofilin is essential for neural tube morphogenesis and neural crest cell migration. Dev Biol.

[B27] Nasarre P, Constantin B, Rouhaud L, Harnois T, Raymond G, Drabkin HA, Bourmeyster N, Roche J (2003). Semaphorin SEMA3F and VEGF have opposing effects on cell attachment and spreading. Neoplasia.

[B28] Goll DE, Thompson VF, Li H, Wei W, Cong J (2003). The calpain system. Physiol Rev.

[B29] Mehendale HM, Limaye PB (2005). Calpain: a death protein that mediates progression of liver injury. Trends Pharmacol Sci.

[B30] Dourdin N, Bhatt AK, Dutt P, Greer PA, Arthur JS, Elce JS, Huttenlocher A (2001). Reduced cell migration and disruption of the actin cytoskeleton in calpain-deficient embryonic fibroblasts. J Biol Chem.

[B31] Arthur JS, Mykles DL (2000). Calpain zymography with casein or fluorescein isothiocyanate casein. Methods Mol Biol.

[B32] Zimmerman UJ, Boring L, Pak JH, Mukerjee N, Wang KK (2000). The calpain small subunit gene is essential: its inactivation results in embryonic lethality. IUBMB Life.

[B33] Larue L, Ohsugi M, Hirchenhain J, Kemler R (1994). E-cadherin null mutant embryos fail to form a trophectoderm epithelium. Proc Natl Acad Sci USA.

[B34] Neuhoff H, Sassoe-Pognetto M, Panzanelli P, Maas C, Witke W, Kneussel M (2005). The actin-binding protein profilin I is localized at synaptic sites in an activity-regulated manner. Eur J Neurosci.

[B35] Nelson WJ, Nusse R (2004). Convergence of Wnt, beta-catenin, and cadherin pathways. Science.

[B36] Schneider VA, Mercola M (2001). Wnt antagonism initiates cardiogenesis in Xenopus laevis. Genes Dev.

[B37] Terami H, Hidaka K, Katsumata T, Iio A, Morisaki T (2004). Wnt11 facilitates embryonic stem cell differentiation to Nkx2.5-positive cardiomyocytes. Biochem Biophys Res Commun.

[B38] Nakamura T, Sano M, Songyang Z, Schneider MD (2003). A Wnt- and beta-catenin-dependent pathway for mammalian cardiac myogenesis. Proc Natl Acad Sci USA.

[B39] De Calisto J, Araya C, Marchant L, Riaz CF, Mayor R (2005). Essential role of non-canonical Wnt signalling in neural crest migration. Development.

[B40] Rousset R, Mack JA, Wharton KA, Axelrod JD, Cadigan KM, Fish MP, Nusse R, Scott MP (2001). Naked cuticle targets dishevelled to antagonize Wnt signal transduction. Genes Dev.

[B41] Wharton KA, Zimmermann G, Rousset R, Scott MP (2001). Vertebrate proteins related to Drosophila Naked Cuticle bind Dishevelled and antagonize Wnt signaling. Dev Biol.

[B42] Michalak M, Guo L, Robertson M, Lozak M, Opas M (2004). Calreticulin in the heart. Mol Cell Biochem.

[B43] Michalak M, Lynch J, Groenendyk J, Guo L, Robert Parker JM, Opas M (2002). Calreticulin in cardiac development and pathology. Biochim Biophys Acta.

[B44] Risebro CA, Smart N, Dupays L, Breckenridge R, Mohun TJ, Riley PR (2006). Hand1 regulates cardiomyocyte proliferation versus differentiation in the developing heart. Development.

[B45] Morin S, Pozzulo G, Robitaille L, Cross J, Nemer M (2005). MEF2-dependent recruitment of the HAND1 transcription factor results in synergistic activation of target promoters. J Biol Chem.

[B46] Loeken MR (2004). Free radicals and birth defects. J Matern Fetal Neonatal Med.

[B47] Persson B (2001). Prevention of fetal malformation with antioxidants in diabetic pregnancy. Pediatr Res.

[B48] BENJAMINI YH Y J ROY STATIST SOC SER B METHO.

[B49] Piedrahita JA, Oetama B, Bennett GD, van Waes J, Kamen BA, Richardson J, Lacey SW, Anderson RG, Finnell RH (1999). Mice lacking the folic acid-binding protein Folbp1 are defective in early embryonic development. Nat Genet.

[B50] UoTaORN Laboratory Gene Ontology Tree Machine. http://genereg.ornl.gov/gotm/.

